# Women’s experiences of severe postnatal psychiatric illness: a systematic review protocol

**DOI:** 10.1186/s13643-019-1085-6

**Published:** 2019-07-17

**Authors:** Harpreet Kaur Sihre, Anne-Marie Simons, Paramjit Gill, Antje Lindenmeyer, Qulsom Fazil

**Affiliations:** 1 Institute of Applied Health Research, University of Birmingham, Birmingham, Edgbaston B15 2TT UK; 2 Birmingham and Solihull Mental Health Foundation Trust, The Barberry, Birmingham, UK; 3 Warwick Medical School, University of Warwick, Coventry, UK

**Keywords:** Systematic review, Meta-ethnography, Severe postnatal psychiatric illness, Perinatal Mental Health Services, Qualitative research, Protocol

## Abstract

**Background:**

Super diversity has become a twenty-first-century phenomena in the UK. The Five Year Forward View Plan for Mental Health commits to improving access to Perinatal Mental Health services for all new mothers. Existing research indicates various postnatal mental illness aetiologies, traditional practices and beliefs, which are important to explore during medical consultation to achieve a collaborative relationship between the patient and clinician. The study of severe postnatal psychiatric illnesses is well established in the quantitative literature; however, the subjective experiences of mothers with severe psychiatric illnesses after childbirth have been given little attention. The aim of this systematic review is to synthesise the small body of qualitative findings, which will achieve a deeper understanding of mothers’ experiences and understandings. This integration of qualitative data is invaluable in facilitating culturally competent strategies in Western settings and informing future research.

**Methods/design:**

This protocol proposes a systematic review of qualitative literature of severe postnatal psychiatric illnesses, using a meta-ethnography approach following the PRISMA-P guidelines. A comprehensive search strategy will be developed to undertake a systematic search via online databases to identify eligible articles. Studies will be considered for eligibility if they include mothers aged 18 and over who have been diagnosed with a severe psychiatric illness during the postnatal period; present primary data on women’s illness experiences; use qualitative methods. Titles and abstracts will be screened by the primary reviewer to identify potential papers. Two independent reviewers will access and read texts in full and agree on the final list of included studies. Discrepancies will be resolved via consultation with a third independent reviewer. The final list of included studies for review will be methodologically appraised by two independent reviewers using the Critical Appraisal Skills Programme. This systematic review protocol has been registered with the International Prospective Register of Systematic Reviews (PROSPERO): CRD42018093674.

**Discussion:**

To date, no systematic review following a meta-ethnographic approach on qualitative explorations of mothers worldwide diagnosed with severe postnatal psychiatric illnesses has been conducted. An amalgamation of this information enables a deeper understanding of how severe postnatal psychiatric illnesses manifests across cultures. This information is useful when devising culturally competent care.

**Systematic review registration:**

CRD42018093674

**Electronic supplementary material:**

The online version of this article (10.1186/s13643-019-1085-6) contains supplementary material, which is available to authorized users.

## Background

A woman’s transition to motherhood has been reported as a major life event involving challenges in the psychological, social and biological spheres [1]. The postnatal period is defined up to a year after childbirth and is considered to be a time of increased susceptibility to psychiatric illnesses for all new mothers across all cultures [2]. Since the 1980s, there has been an upsurge of clinical and academic research to recognise symptomology, prevalence, prognosis, differential diagnosis, and management strategies to prevent adverse outcomes for mother and baby [3]. It is documented that 0.2% of new mothers will experience postnatal psychosis and 0.2% will experience a serious/complex disorder after childbirth such as psychotic depression, schizophrenia, bipolar disorder, and schizoaffective disorder [4, 5]. Previous systematic review findings have confirmed low global incidence of perinatal psychosis from 0.89 to 2.6 in 1000 women, aligning with the commonly reported prevalence among the general population [6]. Although relatively low, this morbidity is significant from a global health perspective because of the long-standing deleterious consequences that go beyond the mother. The long- and short-term adverse outcomes of a mothers’ severe postnatal psychiatric illness can impact the infant, the partner, and the family relationships [2]. In rare cases, a severe postnatal psychiatric illness can lead to maternal and/or infant mortality [7]. The strongest and most established risk factor for susceptibility for severe postnatal psychiatric illnesses is a history of bipolar disorder or previous severe postpartum episodes [8, 9]. Other risk factors identified include obstetric factors, changes in medication, psychological factors, hormonal factors, immunological factors, sleep deprivation, and genetics factors [7].

A severe postnatal psychiatric illness is considered a psychiatric emergency requiring imminent medical attention and referral to secondary or tertiary care services, such as Perinatal Mental Health services [10]. Despite limited data on intervention programmes, existing reviews have examined admission data, outcomes for mothers and programmes and interventions in Mother and Baby units [11]. Findings conclude that this is a favourable environment for interventions and programmes leading to positive outcomes for postnatal mothers with severe psychiatric illnesses [11]. Additionally, previous systematic reviews have examined the efficacy of interventions to prevent (e.g. lithium) and treat (e.g. electroconvulsive therapy) postnatal psychosis [12]. Nonetheless, due to methodological limitations among included studies reviewed, extensive evidence-based recommendations could not be strongly inferred. Although the quantitative and scientific data is pivotal for clinical and cost-effectiveness, it is equally important that preventative and treatment strategies are implemented appropriately and culturally, meeting the subjective needs and experiences of patients and improve better access to care. The National Institute for Health and Care Excellence (NICE) guidelines highlights the importance of understanding illness from the patient’s perspective. We conducted a scoping search of the literature and identified several qualitative papers that reported women’s subjective experiences and understandings of severe postnatal psychiatric illness. Although studies are sparse, the existing qualitative data reports descriptions of illness experiences and author interpretations. This data is appropriate and informative for a meta-ethnography study to provide a higher level of analysis of the phenomena being researched and re-interpretations of meaning across studies [13]. This method allows a rigorous process for critical examination of several accounts to derive substantive interpretations and combine these elements to form a new whole.

Previous studies suggest that ethnicity and culture may influence a patient’s subjective illness beliefs and perceptions of postnatal psychiatric illnesses and appropriate help-seeking behaviours that go against the biological model of illness [14]. Additionally, studies have recognised non-biological determinants such as psychological, psychosocial and cultural factors that influence the evolution of psychopathology after childbirth, and so it is important to explore the intricacies and myriad of aetiology in the context of the patient’s cultural and social milieu [14]. Therefore, understanding conceptualisations of psychiatric illness through a cultural and social lens is invaluable in reaching collaborative patient-centred diagnoses and management plans enabling the development of culturally appropriate treatment. This will also help reduce barriers to accessing care and treatment and encourage all women across cultures to engage with services, which has been prioritised in developing UK policy such as the Five Year Forward View Plan for Mental Health in an era of super diversity [15]. In the UK, females originate from a range of countries and ethnicities, which include heterogeneous cultures, religions and languages. With higher fertility rates among this super diverse cohort, they are likely to be the main users of Maternity and Mental Health services [16]. Experiences of mental health differ across different cultural contexts, also affecting how and where treatment is sought. Therefore, it is important to consider how Perinatal Mental Health care providers can develop appropriate services to meet the needs of ethnic groups through qualitative research understanding their subjective experiences and understandings [17]. To our knowledge, a worldwide comprehensive synthesis of new mothers’ experiences and understandings of severe postnatal psychiatric illnesses remains absent. The aim of the systematic review is to explore postnatal mothers’ experiences of severe psychiatric illnesses and enable a deeper understanding by extending the interpretative possibilities offered by the small body of literature, using a meta-ethnographic approach. The qualitative review synthesis is to go beyond what is currently offered in the literature and offer new in-depth understandings, insights and directions for future research. This information is an important component in an area of developing evidence-informed government policy and practice in Perinatal Mental Health.

## Methods/design

This systematic review protocol has been registered with the International Prospective Register of Systematic Reviews (PROSPERO): CRD42018093674. This systematic review protocol was developed using the Preferred Reporting Items for Systematic review and Meta-Analysis Protocols 2015 checklist (PRISMA-P) (see Additional file 1) [18]. However, it is important to note that not all items on the PRISMA-P checklist will apply, given the qualitative nature of the study design. When we synthesise and write-up the findings of the final review report, we will adhere to the enhancing transparency in reporting the synthesis of qualitative research (ENTREQ) guidelines [19].

### Study design

Our review will aim to use a meta-ethnography developed by Noblit and Hare (1988) to search, interpret and synthesise the phenomena under investigation [20]. This has been recognised as the most developed and structured method used to synthesise qualitative findings that goes beyond a traditional narrative literature review, offering new interpretation, insights and in-depth meaning of primary data. The seven-phase approach of meta-ethnography includes (1) getting started, (2) deciding what is relevant to the initial interest, (3) reading the studies, (4) determining how the studies are related, (5) translating the studies into one another, (6) synthesising translations, and (7) expressing the synthesis.

#### Phase 1: Study aim

Our systematic review using meta-ethnography will aim to provide a worldwide coherent and comprehensive transcultural approach to understand women’s experiences of their psychiatric illnesses after childbirth, including the psychosocial and cultural contributions to the aetiology of illness.

#### Phase 2: Search and selection strategy

##### Study eligibility criteria

The SPIDER (Sample; Phenomenon of Interest; Design; Evaluation; Research type) criterion for qualitative research will be used in lieu of PICO (Participant; Intervention; Comparison; Outcome), which are more specific to develop eligibility criteria for qualitative research studies [21].

##### Sample

This review will include studies describing the experiences of mothers aged 18 years or above. Mothers can be interviewed during the postnatal period or retrospectively. For the proposed systematic review, the postnatal period is defined as up to a year after childbirth. Recall around childbirth and traumatic life events, such as psychotic illness is generally reported to be excellent [22]. Therefore, mothers do not have to be in the postnatal period when participating in studies.

##### Phenomenon of interest

For the purpose of this systematic review, studies will only be included if they include mothers who have been diagnosed with a severe psychiatric illness during the postnatal period.

##### Design

Studies will only be included if they use qualitative methods such as interviews, focus groups as methods of primary data collection.

##### Evaluation

Studies that present primary data on new mothers’ experiences and understandings of severe postnatal psychiatric illness using qualitative methods of data collection.

##### Research type

Qualitative studies only will be included in this review. Although there may be mixed methods studies available, the data offered from the qualitative component is not in-depth for a meta-ethnography study.

##### Study characteristics

No demographic or geographic restriction will be placed on sample participants or study setting, as it is good practice to be as inclusive as possible. Because of limited resources, results will be restricted to English language publications. Studies published from 2000 to 2018 will be considered to include recent literature.

##### Information sources

Peer-reviewed published articles across the following databases:

- CINAHL
- EMBASE
- OVID (PsychInfo, MEDLINE)
- PubMed
- Web of Science Core Collection

The reference list of all relevant articles will be hand searched by the primary reviewer for any additional papers that may meet eligibility for the review.

##### Search strategy

A reference librarian will be consulted with regards to the design of the search strategy, which will be developed using the SPIDER guidelines for qualitative research. Search terms will incorporate medical subject headings (MeSH), text words and/or keywords (see Table 1). The initial search strategy will be designed for Web of Science Core Collection and adapted to suit individual databases (see Additional file 2).

**Table 1:** Search terms

SPIDER heading	Search terms for Web of Science Core Collection
Sample: mothers	“female” OR “females” OR “mum” OR “mums” OR “mother” OR “mothers” OR “women” OR “woman” OR “new mothers” AND
Phenomenon of interest: severe psychiatric illness after childbirth	“postnatal” OR “postpartum” OR “puerperal” OR “perinatal” OR “childbirth” OR “after birth” OR “post-delivery” OR “post neonatal” OR “peripartum” OR “motherhood” OR “after childbirth” AND “psychosis” OR “schizophrenia” OR “bipolar disorder” OR “mania” OR “psychotic episode” OR “severe depression” OR “schizoaffective” OR “mood disorder” AND
Design: qualitative research	“qualitative research” OR “qualitative study” OR “interviews” OR “surveys” OR “focus groups” OR “grounded theory” OR “ethnography” OR “phenomenology” OR “content analysis” OR “thematic analysis” OR “observation” OR “narrative” OR “stories” AND
Evaluation:	“experience” OR “lived experience” OR “attitude” OR “belief” OR “understanding” OR “perceptions” OR “knowledge” OR “view” OR “feeling” OR “opinion” AND
Research type: qualitative or mixed methods	“qualitative research” OR “qualitative analysis” OR “qualitative mixed methods”

##### Selection process

A flow diagram following the PRISMA guidelines will be used in reporting the selection process and all results. Studies will be recorded and managed using data management software such as Endnote. The predetermined search terms will be entered manually across all online database and combined with Boolean terms “and” or “or”. The primary reviewer will run the initial searches across all online databases to identify empirical articles in the field. All titles and abstracts will be screened by the primary reviewer against the eligibility criteria and any potential papers for inclusion will be marked and imported into Endnote. Two independent reviewers will conduct a full-text review of provisionally included studies. Studies will be included or excluded with reason against the predetermined eligibility criteria. Any discrepancies will be resolved through consultation of a third reviewer. If multiple papers are found that describe the same data set, we will include the paper that describes the most comprehensive findings. All reviewers will agree on the final list of included papers.

##### Quality assessment of included studies

Two independent reviewers will critically appraise the final list of included studies using the Critical Appraisal Skills Programme tool [23]. Discussion will be used to resolve discrepancies and potential intervention of a third reviewer to reach consensus. Poor quality studies will be excluded from review to ensure the validity of study findings and trustworthiness of clinical implications. A ‘good’ study will include sufficient original data or at least some interpretive analysis for a meta-ethnography.

#### Phases 3–7: Synthesis of findings

Meta-ethnography offers rigorous synthesising of qualitative research offering a range of depth of meanings, experiences and perspectives of participants across varied contexts. The studies included for review will be read and re-read by an independent reviewer. Reading studies repetitively is central to meta-ethnography as it allows the reviewer to familiarise themselves with the data that will be used for analysis. Items will be extracted and documented in a standardised Microsoft Excel spreadsheet. Data items will include, but not be limited to

- Study title
- Author(s)
- Year of publication
- Country and country income group
- Study design
- Study aim(s)
- Sample and sampling method (including age range)
- Sample characteristics (including ethnicity)
- Setting(s)
- Measurement(s) used for the diagnosis of severe postnatal psychiatric illness
- Data collection and data analysis method(s)
- Key findings (first-order constructs, including any mentions of culture, religion, traditions or postnatal practices).

Meta-ethnographies are often described in terms of first-order, second-order and third-order constructs. First-order constructs reflect the descriptive narratives of the participants reported in the primary studies that relates to our main research inquiry. Any information that falls outside the remit of our research inquiry will not be included to remain close to the phenomena of interest. Although first-order concepts will come from the results section of the paper, when reading and re-reading the studies, if we find original data being discussed in the discussion section of an article, this will also be included as first-order concepts. Second-order constructs are authors’ explanations and interpretations of the data extracts/key concepts, which are usually found in the discussion section of an article. The third-order construct is a synthesis of both the first and second order constructs conducted by the reviewer and team, which begins the development of the line of argument [20]. The next stage of analysis and synthesis involves a translational process to continue the formation of the line of argument across papers. Concepts from each paper will be compared to check for similarities (reciprocal translation) and different accounts between studies will also be highlighted (refutational translation). This will form conceptual categories that reflect the original data from the included papers. The reviewers will be able to make sense of the studies as a whole rather than in isolation, which will determine a reciprocal or refutational synthesis from which a line of argument can be shaped. The primary reviewer will be conducting the analysis with discussion with the second reviewer and academic supervisors to triangulate second and third order concepts (co-authors). Triangulation is a strategy used in the qualitative analysis that allows several perspectives to reach consensus in the themes generated, increasing the validity and convincibility of the overall review findings [24]. Face-to-face and telephone discussions with a second reviewer and academic supervisors will facilitate the translation process and assist with the conceptual innovation. Peer debriefing will add rigour to the analysis and synthesis process adding trustworthiness to the study results.

The data will be managed using Microsoft word documents. First-order, second-order and third-order concepts will be tabulated. A column will be given for each study. Similarities and differences between concepts will be highlighted and colour coordinated. The synthesis will be presented in textual format with the use of a visual diagrams and tables.

The first author has previous experience in conducting a qualitative systematic review using meta-ethnography, which aimed to explore women’s beliefs about the causes of postnatal depression (under review). Academic supervisors who will be involved in the translational process are also experienced in conducting meta-ethnographies and qualitative research. The first author is currently completing a Ph.D. using Interpretative Phenomenological Analyses and so has experience in using complex methods for analysing qualitative data.

## Discussion

A worldwide comprehensive synthesis of qualitative primary data on mothers’ experiences of severe psychiatric illness after childbirth is deficient in the current literature. Our proposed review will enable a deeper understanding and interpretation of new mothers’ experiences, which is vital in informing a patient-centred care approach within a super diverse setting. The failure of providing culturally competent health care services may have a negative impact on health care interactions and outcomes.

### Limitations and strengths

Because of limited resources, the review will only include studies in the English language. Studies published in other languages may contain useful information for the review, which may prove invaluable given the international focus of this review. Furthermore, studies in non-English languages may provide various conceptualisations of postnatal psychiatric illnesses from a cultural angle, which may prove invaluable given the international focus of this review.

The major strength of this review will be the use of a systematic and transparent approach, employing validated and recommended tools. The quality of the review will be strengthened by the involvement of multiple reviewers for validation of study selection and critical appraisal. The inclusion of a second reviewer and academic supervisors to triangulate concepts, interpretations and overall review findings will make sure the findings are robust, comprehensive and well-developed ensuring the credibility and validity of the synthesis and findings. We will only include studies with a strong critical appraisal score, thus adding to the trustworthiness of review findings and clinical implications.

## Additional files

Additional file 1: Preferred Reporting Items for Systematic review and Meta-Analysis Protocols 2015 checklist (PRISMA-P). (DOCX 29 kb)

Additional file 2: Search strategy. Web of Science Core Collection. (DOCX 155 kb)

## Data Availability

Not applicable.

## Abbreviations

CASP: Critical Appraisal Skills Programme
CINAHL: The Cumulative Index to Nursing and Allied Health Literature
PND: Postnatal depression
PRISMA: Preferred Reporting Items for Systematic Reviews and Meta-Analyses
PROSPERO: International prospective register of systematic reviews
SPIDER: Sample, Phenomenon of Interest, Design, Evaluation, Research type
UK: United Kingdom
