# Supplementary material for: Women’s experiences of severe postnatal psychiatric illness: a systematic review protocol
Source: Syst Rev. 2019 Jul 17;8:173. doi: 10.1186/s13643-019-1085-6 (PMC6637608; doi:10.1186/s13643-019-1085-6)
Supplement: Supplementary file 2 — Search strategy. Web of Science Core Collection. (DOCX 155 kb) [file 13643_2019_1085_MOESM2_ESM.docx]

**Appendix 1.** Search strategy. Web of Science Core Collection.

| Search History | | | | | |
| --- | --- | --- | --- | --- | --- |
| **Set** | **Results** | **Save History / Create AlertOpen Saved History** | **Edit Sets** | **Combine Sets**  ** AND   OR**  **Combine** | **Delete Sets**  **Select All  Delete** |
|  | | | | | |
| # 60 | [**36**](http://apps.webofknowledge.com/summary.do?product=WOS&doc=1&qid=60&SID=C56HEtGVniJkJ8gFEJW&search_mode=CombineSearches&update_back2search_link_param=yes) | #59 AND #56 AND #45 AND #31 AND #22 AND #10  *Indexes=SCI-EXPANDED, SSCI, A&HCI, CPCI-S, CPCI-SSH, BKCI-S, BKCI-SSH, ESCI, CCR-EXPANDED, IC Timespan=All years* | [Edit](http://apps.webofknowledge.com/WOS_AdvancedSearch_input.do?product=WOS&SID=C56HEtGVniJkJ8gFEJW&search_mode=AdvancedSearch&replaceSetId=60&editState=init) |  |  |
|  | | | | | |
| # 59 | [**184,710**](http://apps.webofknowledge.com/summary.do?product=WOS&doc=1&qid=59&SID=C56HEtGVniJkJ8gFEJW&search_mode=CombineSearches&update_back2search_link_param=yes) | #58 OR #57  *Indexes=SCI-EXPANDED, SSCI, A&HCI, CPCI-S, CPCI-SSH, BKCI-S, BKCI-SSH, ESCI, CCR-EXPANDED, IC Timespan=All years* | [Edit](http://apps.webofknowledge.com/WOS_AdvancedSearch_input.do?product=WOS&SID=C56HEtGVniJkJ8gFEJW&search_mode=AdvancedSearch&replaceSetId=59&editState=init) |  |  |
|  | | | | | |
| # 58 | [**16,394**](http://apps.webofknowledge.com/summary.do?product=WOS&doc=1&qid=58&SID=C56HEtGVniJkJ8gFEJW&search_mode=GeneralSearch&update_back2search_link_param=yes) | **TOPIC:** (qualitative mixed methods)  *Indexes=SCI-EXPANDED, SSCI, A&HCI, CPCI-S, CPCI-SSH, BKCI-S, BKCI-SSH, ESCI, CCR-EXPANDED, IC Timespan=All years* | [Edit](http://apps.webofknowledge.com/WOS_AdvancedSearch_input.do?product=WOS&SID=C56HEtGVniJkJ8gFEJW&search_mode=AdvancedSearch&replaceSetId=58&editState=init) |  |  |
|  | | | | | |
| # 57 | [**176,726**](http://apps.webofknowledge.com/summary.do?product=WOS&doc=1&qid=57&SID=C56HEtGVniJkJ8gFEJW&search_mode=GeneralSearch&update_back2search_link_param=yes) | **TOPIC:** (qualitative analysis)  *Indexes=SCI-EXPANDED, SSCI, A&HCI, CPCI-S, CPCI-SSH, BKCI-S, BKCI-SSH, ESCI, CCR-EXPANDED, IC Timespan=All years* | [Edit](http://apps.webofknowledge.com/WOS_AdvancedSearch_input.do?product=WOS&SID=C56HEtGVniJkJ8gFEJW&search_mode=AdvancedSearch&replaceSetId=57&editState=init) |  |  |
|  | | | | | |
| # 56 | [**6,137,321**](http://apps.webofknowledge.com/summary.do?product=WOS&doc=1&qid=56&SID=C56HEtGVniJkJ8gFEJW&search_mode=CombineSearches&update_back2search_link_param=yes) | #55 OR #54 OR #53 OR #52 OR #51 OR #50 OR #49 OR #48 OR #47 OR #46  *Indexes=SCI-EXPANDED, SSCI, A&HCI, CPCI-S, CPCI-SSH, BKCI-S, BKCI-SSH, ESCI, CCR-EXPANDED, IC Timespan=All years* | [Edit](http://apps.webofknowledge.com/WOS_AdvancedSearch_input.do?product=WOS&SID=C56HEtGVniJkJ8gFEJW&search_mode=AdvancedSearch&replaceSetId=56&editState=init) |  |  |
|  | | | | | |
| # 55 | [**160,057**](http://apps.webofknowledge.com/summary.do?product=WOS&doc=1&qid=55&SID=C56HEtGVniJkJ8gFEJW&search_mode=GeneralSearch&update_back2search_link_param=yes) | **TOPIC:** (opinion)  *Indexes=SCI-EXPANDED, SSCI, A&HCI, CPCI-S, CPCI-SSH, BKCI-S, BKCI-SSH, ESCI, CCR-EXPANDED, IC Timespan=All years* | [Edit](http://apps.webofknowledge.com/WOS_AdvancedSearch_input.do?product=WOS&SID=C56HEtGVniJkJ8gFEJW&search_mode=AdvancedSearch&replaceSetId=55&editState=init) |  |  |
|  | | | | | |
| # 54 | [**179,779**](http://apps.webofknowledge.com/summary.do?product=WOS&doc=1&qid=54&SID=C56HEtGVniJkJ8gFEJW&search_mode=GeneralSearch&update_back2search_link_param=yes) | **TOPIC:** (feeling)  *Indexes=SCI-EXPANDED, SSCI, A&HCI, CPCI-S, CPCI-SSH, BKCI-S, BKCI-SSH, ESCI, CCR-EXPANDED, IC Timespan=All years* | [Edit](http://apps.webofknowledge.com/WOS_AdvancedSearch_input.do?product=WOS&SID=C56HEtGVniJkJ8gFEJW&search_mode=AdvancedSearch&replaceSetId=54&editState=init) |  |  |
|  | | | | | |
| # 53 | [**957,537**](http://apps.webofknowledge.com/summary.do?product=WOS&doc=1&qid=53&SID=C56HEtGVniJkJ8gFEJW&search_mode=GeneralSearch&update_back2search_link_param=yes) | **TOPIC:** (view)  *Indexes=SCI-EXPANDED, SSCI, A&HCI, CPCI-S, CPCI-SSH, BKCI-S, BKCI-SSH, ESCI, CCR-EXPANDED, IC Timespan=All years* | [Edit](http://apps.webofknowledge.com/WOS_AdvancedSearch_input.do?product=WOS&SID=C56HEtGVniJkJ8gFEJW&search_mode=AdvancedSearch&replaceSetId=53&editState=init) |  |  |
|  | | | | | |
| # 52 | [**1,268,486**](http://apps.webofknowledge.com/summary.do?product=WOS&doc=1&qid=52&SID=C56HEtGVniJkJ8gFEJW&search_mode=GeneralSearch&update_back2search_link_param=yes) | **TOPIC:** (knowledge)  *Indexes=SCI-EXPANDED, SSCI, A&HCI, CPCI-S, CPCI-SSH, BKCI-S, BKCI-SSH, ESCI, CCR-EXPANDED, IC Timespan=All years* | [Edit](http://apps.webofknowledge.com/WOS_AdvancedSearch_input.do?product=WOS&SID=C56HEtGVniJkJ8gFEJW&search_mode=AdvancedSearch&replaceSetId=52&editState=init) |  |  |
|  | | | | | |
| # 51 | [**539,214**](http://apps.webofknowledge.com/summary.do?product=WOS&doc=1&qid=51&SID=C56HEtGVniJkJ8gFEJW&search_mode=GeneralSearch&update_back2search_link_param=yes) | **TOPIC:** (perceptions)  *Indexes=SCI-EXPANDED, SSCI, A&HCI, CPCI-S, CPCI-SSH, BKCI-S, BKCI-SSH, ESCI, CCR-EXPANDED, IC Timespan=All years* | [Edit](http://apps.webofknowledge.com/WOS_AdvancedSearch_input.do?product=WOS&SID=C56HEtGVniJkJ8gFEJW&search_mode=AdvancedSearch&replaceSetId=51&editState=init) |  |  |
|  | | | | | |
| # 50 | [**2,200,824**](http://apps.webofknowledge.com/summary.do?product=WOS&doc=1&qid=50&SID=C56HEtGVniJkJ8gFEJW&search_mode=GeneralSearch&update_back2search_link_param=yes) | **TOPIC:** (understanding)  *Indexes=SCI-EXPANDED, SSCI, A&HCI, CPCI-S, CPCI-SSH, BKCI-S, BKCI-SSH, ESCI, CCR-EXPANDED, IC Timespan=All years* | [Edit](http://apps.webofknowledge.com/WOS_AdvancedSearch_input.do?product=WOS&SID=C56HEtGVniJkJ8gFEJW&search_mode=AdvancedSearch&replaceSetId=50&editState=init) |  |  |
|  | | | | | |
| # 49 | [**184,229**](http://apps.webofknowledge.com/summary.do?product=WOS&doc=1&qid=49&SID=C56HEtGVniJkJ8gFEJW&search_mode=GeneralSearch&update_back2search_link_param=yes) | **TOPIC:** (belief)  *Indexes=SCI-EXPANDED, SSCI, A&HCI, CPCI-S, CPCI-SSH, BKCI-S, BKCI-SSH, ESCI, CCR-EXPANDED, IC Timespan=All years* | [Edit](http://apps.webofknowledge.com/WOS_AdvancedSearch_input.do?product=WOS&SID=C56HEtGVniJkJ8gFEJW&search_mode=AdvancedSearch&replaceSetId=49&editState=init) |  |  |
|  | | | | | |
| # 48 | [**340,623**](http://apps.webofknowledge.com/summary.do?product=WOS&doc=1&qid=48&SID=C56HEtGVniJkJ8gFEJW&search_mode=GeneralSearch&update_back2search_link_param=yes) | **TOPIC:** (attitude)  *Indexes=SCI-EXPANDED, SSCI, A&HCI, CPCI-S, CPCI-SSH, BKCI-S, BKCI-SSH, ESCI, CCR-EXPANDED, IC Timespan=All years* | [Edit](http://apps.webofknowledge.com/WOS_AdvancedSearch_input.do?product=WOS&SID=C56HEtGVniJkJ8gFEJW&search_mode=AdvancedSearch&replaceSetId=48&editState=init) |  |  |
|  | | | | | |
| # 47 | [**93,462**](http://apps.webofknowledge.com/summary.do?product=WOS&doc=1&qid=47&SID=C56HEtGVniJkJ8gFEJW&search_mode=GeneralSearch&update_back2search_link_param=yes) | **TOPIC:** (lived experience)  *Indexes=SCI-EXPANDED, SSCI, A&HCI, CPCI-S, CPCI-SSH, BKCI-S, BKCI-SSH, ESCI, CCR-EXPANDED, IC Timespan=All years* | [Edit](http://apps.webofknowledge.com/WOS_AdvancedSearch_input.do?product=WOS&SID=C56HEtGVniJkJ8gFEJW&search_mode=AdvancedSearch&replaceSetId=47&editState=init) |  |  |
|  | | | | | |
| # 46 | [**1,646,937**](http://apps.webofknowledge.com/summary.do?product=WOS&doc=1&qid=46&SID=C56HEtGVniJkJ8gFEJW&search_mode=GeneralSearch&update_back2search_link_param=yes) | **TOPIC:** (experience)  *Indexes=SCI-EXPANDED, SSCI, A&HCI, CPCI-S, CPCI-SSH, BKCI-S, BKCI-SSH, ESCI, CCR-EXPANDED, IC Timespan=All years* | [Edit](http://apps.webofknowledge.com/WOS_AdvancedSearch_input.do?product=WOS&SID=C56HEtGVniJkJ8gFEJW&search_mode=AdvancedSearch&replaceSetId=46&editState=init) |  |  |
|  | | | | | |
| # 45 | [**3,854,493**](http://apps.webofknowledge.com/summary.do?product=WOS&doc=1&qid=45&SID=C56HEtGVniJkJ8gFEJW&search_mode=CombineSearches&update_back2search_link_param=yes) | #44 OR #43 OR #42 OR #41 OR #40 OR #39 OR #38 OR #37 OR #36 OR #35 OR #34 OR #33 OR #32  *Indexes=SCI-EXPANDED, SSCI, A&HCI, CPCI-S, CPCI-SSH, BKCI-S, BKCI-SSH, ESCI, CCR-EXPANDED, IC Timespan=All years* | [Edit](http://apps.webofknowledge.com/WOS_AdvancedSearch_input.do?product=WOS&SID=C56HEtGVniJkJ8gFEJW&search_mode=AdvancedSearch&replaceSetId=45&editState=init) |  |  |
|  | | | | | |
| # 44 | [**170,849**](http://apps.webofknowledge.com/summary.do?product=WOS&doc=1&qid=44&SID=C56HEtGVniJkJ8gFEJW&search_mode=GeneralSearch&update_back2search_link_param=yes) | **TOPIC:** (stories)  *Indexes=SCI-EXPANDED, SSCI, A&HCI, CPCI-S, CPCI-SSH, BKCI-S, BKCI-SSH, ESCI, CCR-EXPANDED, IC Timespan=All years* | [Edit](http://apps.webofknowledge.com/WOS_AdvancedSearch_input.do?product=WOS&SID=C56HEtGVniJkJ8gFEJW&search_mode=AdvancedSearch&replaceSetId=44&editState=init) |  |  |
|  | | | | | |
| # 43 | [**136,340**](http://apps.webofknowledge.com/summary.do?product=WOS&doc=1&qid=43&SID=C56HEtGVniJkJ8gFEJW&search_mode=GeneralSearch&update_back2search_link_param=yes) | **TOPIC:** (narrative)  *Indexes=SCI-EXPANDED, SSCI, A&HCI, CPCI-S, CPCI-SSH, BKCI-S, BKCI-SSH, ESCI, CCR-EXPANDED, IC Timespan=All years* | [Edit](http://apps.webofknowledge.com/WOS_AdvancedSearch_input.do?product=WOS&SID=C56HEtGVniJkJ8gFEJW&search_mode=AdvancedSearch&replaceSetId=43&editState=init) |  |  |
|  | | | | | |
| # 42 | [**1,359,490**](http://apps.webofknowledge.com/summary.do?product=WOS&doc=1&qid=42&SID=C56HEtGVniJkJ8gFEJW&search_mode=GeneralSearch&update_back2search_link_param=yes) | **TOPIC:** (observation)  *Indexes=SCI-EXPANDED, SSCI, A&HCI, CPCI-S, CPCI-SSH, BKCI-S, BKCI-SSH, ESCI, CCR-EXPANDED, IC Timespan=All years* | [Edit](http://apps.webofknowledge.com/WOS_AdvancedSearch_input.do?product=WOS&SID=C56HEtGVniJkJ8gFEJW&search_mode=AdvancedSearch&replaceSetId=42&editState=init) |  |  |
|  | | | | | |
| # 41 | [**31,722**](http://apps.webofknowledge.com/summary.do?product=WOS&doc=1&qid=41&SID=C56HEtGVniJkJ8gFEJW&search_mode=GeneralSearch&update_back2search_link_param=yes) | **TOPIC:** (thematic analysis)  *Indexes=SCI-EXPANDED, SSCI, A&HCI, CPCI-S, CPCI-SSH, BKCI-S, BKCI-SSH, ESCI, CCR-EXPANDED, IC Timespan=All years* | [Edit](http://apps.webofknowledge.com/WOS_AdvancedSearch_input.do?product=WOS&SID=C56HEtGVniJkJ8gFEJW&search_mode=AdvancedSearch&replaceSetId=41&editState=init) |  |  |
|  | | | | | |
| You will only be able to save the 40 sets below this line. | | | | | |
|  | | | | | |
| # 40 | [**443,246**](http://apps.webofknowledge.com/summary.do?product=WOS&doc=1&qid=40&SID=C56HEtGVniJkJ8gFEJW&search_mode=GeneralSearch&update_back2search_link_param=yes) | **TOPIC:** (content analysis)  *Indexes=SCI-EXPANDED, SSCI, A&HCI, CPCI-S, CPCI-SSH, BKCI-S, BKCI-SSH, ESCI, CCR-EXPANDED, IC Timespan=All years* | [Edit](http://apps.webofknowledge.com/WOS_AdvancedSearch_input.do?product=WOS&SID=C56HEtGVniJkJ8gFEJW&search_mode=AdvancedSearch&replaceSetId=40&editState=init) |  |  |
|  | | | | | |
| # 39 | [**38,311**](http://apps.webofknowledge.com/summary.do?product=WOS&doc=1&qid=39&SID=C56HEtGVniJkJ8gFEJW&search_mode=GeneralSearch&update_back2search_link_param=yes) | **TOPIC:** (phenomenology)  *Indexes=SCI-EXPANDED, SSCI, A&HCI, CPCI-S, CPCI-SSH, BKCI-S, BKCI-SSH, ESCI, CCR-EXPANDED, IC Timespan=All years* | [Edit](http://apps.webofknowledge.com/WOS_AdvancedSearch_input.do?product=WOS&SID=C56HEtGVniJkJ8gFEJW&search_mode=AdvancedSearch&replaceSetId=39&editState=init) |  |  |
|  | | | | | |
| # 38 | [**21,399**](http://apps.webofknowledge.com/summary.do?product=WOS&doc=1&qid=38&SID=C56HEtGVniJkJ8gFEJW&search_mode=GeneralSearch&update_back2search_link_param=yes) | **TOPIC:** (ethnography)  *Indexes=SCI-EXPANDED, SSCI, A&HCI, CPCI-S, CPCI-SSH, BKCI-S, BKCI-SSH, ESCI, CCR-EXPANDED, IC Timespan=All years* | [Edit](http://apps.webofknowledge.com/WOS_AdvancedSearch_input.do?product=WOS&SID=C56HEtGVniJkJ8gFEJW&search_mode=AdvancedSearch&replaceSetId=38&editState=init) |  |  |
|  | | | | | |
| # 37 | [**81,312**](http://apps.webofknowledge.com/summary.do?product=WOS&doc=1&qid=37&SID=C56HEtGVniJkJ8gFEJW&search_mode=GeneralSearch&update_back2search_link_param=yes) | **TOPIC:** (grounded theory)  *Indexes=SCI-EXPANDED, SSCI, A&HCI, CPCI-S, CPCI-SSH, BKCI-S, BKCI-SSH, ESCI, CCR-EXPANDED, IC Timespan=All years* | [Edit](http://apps.webofknowledge.com/WOS_AdvancedSearch_input.do?product=WOS&SID=C56HEtGVniJkJ8gFEJW&search_mode=AdvancedSearch&replaceSetId=37&editState=init) |  |  |
|  | | | | | |
| # 36 | [**248,607**](http://apps.webofknowledge.com/summary.do?product=WOS&doc=1&qid=36&SID=C56HEtGVniJkJ8gFEJW&search_mode=GeneralSearch&update_back2search_link_param=yes) | **TOPIC:** (focus groups)  *Indexes=SCI-EXPANDED, SSCI, A&HCI, CPCI-S, CPCI-SSH, BKCI-S, BKCI-SSH, ESCI, CCR-EXPANDED, IC Timespan=All years* | [Edit](http://apps.webofknowledge.com/WOS_AdvancedSearch_input.do?product=WOS&SID=C56HEtGVniJkJ8gFEJW&search_mode=AdvancedSearch&replaceSetId=36&editState=init) |  |  |
|  | | | | | |
| # 35 | [**1,100,522**](http://apps.webofknowledge.com/summary.do?product=WOS&doc=1&qid=35&SID=C56HEtGVniJkJ8gFEJW&search_mode=GeneralSearch&update_back2search_link_param=yes) | **TOPIC:** (surveys)  *Indexes=SCI-EXPANDED, SSCI, A&HCI, CPCI-S, CPCI-SSH, BKCI-S, BKCI-SSH, ESCI, CCR-EXPANDED, IC Timespan=All years* | [Edit](http://apps.webofknowledge.com/WOS_AdvancedSearch_input.do?product=WOS&SID=C56HEtGVniJkJ8gFEJW&search_mode=AdvancedSearch&replaceSetId=35&editState=init) |  |  |
|  | | | | | |
| # 34 | [**465,881**](http://apps.webofknowledge.com/summary.do?product=WOS&doc=1&qid=34&SID=C56HEtGVniJkJ8gFEJW&search_mode=GeneralSearch&update_back2search_link_param=yes) | **TOPIC:** (interviews)  *Indexes=SCI-EXPANDED, SSCI, A&HCI, CPCI-S, CPCI-SSH, BKCI-S, BKCI-SSH, ESCI, CCR-EXPANDED, IC Timespan=All years* | [Edit](http://apps.webofknowledge.com/WOS_AdvancedSearch_input.do?product=WOS&SID=C56HEtGVniJkJ8gFEJW&search_mode=AdvancedSearch&replaceSetId=34&editState=init) |  |  |
|  | | | | | |
| # 33 | [**247,375**](http://apps.webofknowledge.com/summary.do?product=WOS&doc=1&qid=33&SID=C56HEtGVniJkJ8gFEJW&search_mode=GeneralSearch&update_back2search_link_param=yes) | **TOPIC:** (qualitative study)  *Indexes=SCI-EXPANDED, SSCI, A&HCI, CPCI-S, CPCI-SSH, BKCI-S, BKCI-SSH, ESCI, CCR-EXPANDED, IC Timespan=All years* | [Edit](http://apps.webofknowledge.com/WOS_AdvancedSearch_input.do?product=WOS&SID=C56HEtGVniJkJ8gFEJW&search_mode=AdvancedSearch&replaceSetId=33&editState=init) |  |  |
|  | | | | | |
| # 32 | [**128,466**](http://apps.webofknowledge.com/summary.do?product=WOS&doc=1&qid=32&SID=C56HEtGVniJkJ8gFEJW&search_mode=GeneralSearch&update_back2search_link_param=yes) | **TOPIC:** (qualitative research)  *Indexes=SCI-EXPANDED, SSCI, A&HCI, CPCI-S, CPCI-SSH, BKCI-S, BKCI-SSH, ESCI, CCR-EXPANDED, IC Timespan=All years* | [Edit](http://apps.webofknowledge.com/WOS_AdvancedSearch_input.do?product=WOS&SID=C56HEtGVniJkJ8gFEJW&search_mode=AdvancedSearch&replaceSetId=32&editState=init) |  |  |
|  | | | | | |
| # 31 | [**296,954**](http://apps.webofknowledge.com/summary.do?product=WOS&doc=1&qid=31&SID=C56HEtGVniJkJ8gFEJW&search_mode=CombineSearches&update_back2search_link_param=yes) | #30 OR #29 OR #28 OR #27 OR #26 OR #25 OR #24 OR #23  *Indexes=SCI-EXPANDED, SSCI, A&HCI, CPCI-S, CPCI-SSH, BKCI-S, BKCI-SSH, ESCI, CCR-EXPANDED, IC Timespan=All years* | [Edit](http://apps.webofknowledge.com/WOS_AdvancedSearch_input.do?product=WOS&SID=C56HEtGVniJkJ8gFEJW&search_mode=AdvancedSearch&replaceSetId=31&editState=init) |  |  |
|  | | | | | |
| # 30 | [**51,351**](http://apps.webofknowledge.com/summary.do?product=WOS&doc=1&qid=30&SID=C56HEtGVniJkJ8gFEJW&search_mode=GeneralSearch&update_back2search_link_param=yes) | **TOPIC:** (mood disorder)  *Indexes=SCI-EXPANDED, SSCI, A&HCI, CPCI-S, CPCI-SSH, BKCI-S, BKCI-SSH, ESCI, CCR-EXPANDED, IC Timespan=All years* | [Edit](http://apps.webofknowledge.com/WOS_AdvancedSearch_input.do?product=WOS&SID=C56HEtGVniJkJ8gFEJW&search_mode=AdvancedSearch&replaceSetId=30&editState=init) |  |  |
|  | | | | | |
| # 29 | [**7,671**](http://apps.webofknowledge.com/summary.do?product=WOS&doc=1&qid=29&SID=C56HEtGVniJkJ8gFEJW&search_mode=GeneralSearch&update_back2search_link_param=yes) | **TOPIC:** (schizoaffective)  *Indexes=SCI-EXPANDED, SSCI, A&HCI, CPCI-S, CPCI-SSH, BKCI-S, BKCI-SSH, ESCI, CCR-EXPANDED, IC Timespan=All years* | [Edit](http://apps.webofknowledge.com/WOS_AdvancedSearch_input.do?product=WOS&SID=C56HEtGVniJkJ8gFEJW&search_mode=AdvancedSearch&replaceSetId=29&editState=init) |  |  |
|  | | | | | |
| # 28 | [**27,559**](http://apps.webofknowledge.com/summary.do?product=WOS&doc=1&qid=28&SID=C56HEtGVniJkJ8gFEJW&search_mode=GeneralSearch&update_back2search_link_param=yes) | **TOPIC:** (severe depression)  *Indexes=SCI-EXPANDED, SSCI, A&HCI, CPCI-S, CPCI-SSH, BKCI-S, BKCI-SSH, ESCI, CCR-EXPANDED, IC Timespan=All years* | [Edit](http://apps.webofknowledge.com/WOS_AdvancedSearch_input.do?product=WOS&SID=C56HEtGVniJkJ8gFEJW&search_mode=AdvancedSearch&replaceSetId=28&editState=init) |  |  |
|  | | | | | |
| # 27 | [**6,516**](http://apps.webofknowledge.com/summary.do?product=WOS&doc=1&qid=27&SID=C56HEtGVniJkJ8gFEJW&search_mode=GeneralSearch&update_back2search_link_param=yes) | **TOPIC:** (psychotic episode)  *Indexes=SCI-EXPANDED, SSCI, A&HCI, CPCI-S, CPCI-SSH, BKCI-S, BKCI-SSH, ESCI, CCR-EXPANDED, IC Timespan=All years* | [Edit](http://apps.webofknowledge.com/WOS_AdvancedSearch_input.do?product=WOS&SID=C56HEtGVniJkJ8gFEJW&search_mode=AdvancedSearch&replaceSetId=27&editState=init) |  |  |
|  | | | | | |
| # 26 | [**14,686**](http://apps.webofknowledge.com/summary.do?product=WOS&doc=1&qid=26&SID=C56HEtGVniJkJ8gFEJW&search_mode=GeneralSearch&update_back2search_link_param=yes) | **TOPIC:** (mania)  *Indexes=SCI-EXPANDED, SSCI, A&HCI, CPCI-S, CPCI-SSH, BKCI-S, BKCI-SSH, ESCI, CCR-EXPANDED, IC Timespan=All years* | [Edit](http://apps.webofknowledge.com/WOS_AdvancedSearch_input.do?product=WOS&SID=C56HEtGVniJkJ8gFEJW&search_mode=AdvancedSearch&replaceSetId=26&editState=init) |  |  |
|  | | | | | |
| # 25 | [**53,889**](http://apps.webofknowledge.com/summary.do?product=WOS&doc=1&qid=25&SID=C56HEtGVniJkJ8gFEJW&search_mode=GeneralSearch&update_back2search_link_param=yes) | **TOPIC:** (bipolar disorder)  *Indexes=SCI-EXPANDED, SSCI, A&HCI, CPCI-S, CPCI-SSH, BKCI-S, BKCI-SSH, ESCI, CCR-EXPANDED, IC Timespan=All years* | [Edit](http://apps.webofknowledge.com/WOS_AdvancedSearch_input.do?product=WOS&SID=C56HEtGVniJkJ8gFEJW&search_mode=AdvancedSearch&replaceSetId=25&editState=init) |  |  |
|  | | | | | |
| # 24 | [**169,163**](http://apps.webofknowledge.com/summary.do?product=WOS&doc=1&qid=24&SID=C56HEtGVniJkJ8gFEJW&search_mode=GeneralSearch&update_back2search_link_param=yes) | **TOPIC:** (schizophrenia)  *Indexes=SCI-EXPANDED, SSCI, A&HCI, CPCI-S, CPCI-SSH, BKCI-S, BKCI-SSH, ESCI, CCR-EXPANDED, IC Timespan=All years* | [Edit](http://apps.webofknowledge.com/WOS_AdvancedSearch_input.do?product=WOS&SID=C56HEtGVniJkJ8gFEJW&search_mode=AdvancedSearch&replaceSetId=24&editState=init) |  |  |
|  | | | | | |
| # 23 | [**59,650**](http://apps.webofknowledge.com/summary.do?product=WOS&doc=1&qid=23&SID=C56HEtGVniJkJ8gFEJW&search_mode=GeneralSearch&update_back2search_link_param=yes) | **TOPIC:** (psychosis)  *Indexes=SCI-EXPANDED, SSCI, A&HCI, CPCI-S, CPCI-SSH, BKCI-S, BKCI-SSH, ESCI, CCR-EXPANDED, IC Timespan=All years* | [Edit](http://apps.webofknowledge.com/WOS_AdvancedSearch_input.do?product=WOS&SID=C56HEtGVniJkJ8gFEJW&search_mode=AdvancedSearch&replaceSetId=23&editState=init) |  |  |
|  | | | | | |
| # 22 | [**306,625**](http://apps.webofknowledge.com/summary.do?product=WOS&doc=1&qid=22&SID=C56HEtGVniJkJ8gFEJW&search_mode=CombineSearches&update_back2search_link_param=yes) | #21 OR #20 OR #19 OR #18 OR #17 OR #16 OR #15 OR #14 OR #13 OR #12 OR #11  *Indexes=SCI-EXPANDED, SSCI, A&HCI, CPCI-S, CPCI-SSH, BKCI-S, BKCI-SSH, ESCI, CCR-EXPANDED, IC Timespan=All years* | [Edit](http://apps.webofknowledge.com/WOS_AdvancedSearch_input.do?product=WOS&SID=C56HEtGVniJkJ8gFEJW&search_mode=AdvancedSearch&replaceSetId=22&editState=init) |  |  |
|  | | | | | |
| # 21 | [**5,703**](http://apps.webofknowledge.com/summary.do?product=WOS&doc=1&qid=21&SID=C56HEtGVniJkJ8gFEJW&search_mode=GeneralSearch&update_back2search_link_param=yes) | **TOPIC:** (after childbirth)  *Indexes=SCI-EXPANDED, SSCI, A&HCI, CPCI-S, CPCI-SSH, BKCI-S, BKCI-SSH, ESCI, CCR-EXPANDED, IC Timespan=All years* | [Edit](http://apps.webofknowledge.com/WOS_AdvancedSearch_input.do?product=WOS&SID=C56HEtGVniJkJ8gFEJW&search_mode=AdvancedSearch&replaceSetId=21&editState=init) |  |  |
|  | | | | | |
| # 20 | [**9,941**](http://apps.webofknowledge.com/summary.do?product=WOS&doc=1&qid=20&SID=C56HEtGVniJkJ8gFEJW&search_mode=GeneralSearch&update_back2search_link_param=yes) | **TOPIC:** (motherhood)  *Indexes=SCI-EXPANDED, SSCI, A&HCI, CPCI-S, CPCI-SSH, BKCI-S, BKCI-SSH, ESCI, CCR-EXPANDED, IC Timespan=All years* | [Edit](http://apps.webofknowledge.com/WOS_AdvancedSearch_input.do?product=WOS&SID=C56HEtGVniJkJ8gFEJW&search_mode=AdvancedSearch&replaceSetId=20&editState=init) |  |  |
|  | | | | | |
| # 19 | [**4,899**](http://apps.webofknowledge.com/summary.do?product=WOS&doc=1&qid=19&SID=C56HEtGVniJkJ8gFEJW&search_mode=GeneralSearch&update_back2search_link_param=yes) | **TOPIC:** (peripartum)  *Indexes=SCI-EXPANDED, SSCI, A&HCI, CPCI-S, CPCI-SSH, BKCI-S, BKCI-SSH, ESCI, CCR-EXPANDED, IC Timespan=All years* | [Edit](http://apps.webofknowledge.com/WOS_AdvancedSearch_input.do?product=WOS&SID=C56HEtGVniJkJ8gFEJW&search_mode=AdvancedSearch&replaceSetId=19&editState=init) |  |  |
|  | | | | | |
| # 18 | [**8,340**](http://apps.webofknowledge.com/summary.do?product=WOS&doc=1&qid=18&SID=C56HEtGVniJkJ8gFEJW&search_mode=GeneralSearch&update_back2search_link_param=yes) | **TOPIC:** (post neonatal)  *Indexes=SCI-EXPANDED, SSCI, A&HCI, CPCI-S, CPCI-SSH, BKCI-S, BKCI-SSH, ESCI, CCR-EXPANDED, IC Timespan=All years* | [Edit](http://apps.webofknowledge.com/WOS_AdvancedSearch_input.do?product=WOS&SID=C56HEtGVniJkJ8gFEJW&search_mode=AdvancedSearch&replaceSetId=18&editState=init) |  |  |
|  | | | | | |
| # 17 | [**980**](http://apps.webofknowledge.com/summary.do?product=WOS&doc=1&qid=17&SID=C56HEtGVniJkJ8gFEJW&search_mode=GeneralSearch&update_back2search_link_param=yes) | **TOPIC:** (post-delivery)  *Indexes=SCI-EXPANDED, SSCI, A&HCI, CPCI-S, CPCI-SSH, BKCI-S, BKCI-SSH, ESCI, CCR-EXPANDED, IC Timespan=All years* | [Edit](http://apps.webofknowledge.com/WOS_AdvancedSearch_input.do?product=WOS&SID=C56HEtGVniJkJ8gFEJW&search_mode=AdvancedSearch&replaceSetId=17&editState=init) |  |  |
|  | | | | | |
| # 16 | [**90,953**](http://apps.webofknowledge.com/summary.do?product=WOS&doc=1&qid=16&SID=C56HEtGVniJkJ8gFEJW&search_mode=GeneralSearch&update_back2search_link_param=yes) | **TOPIC:** (after birth)  *Indexes=SCI-EXPANDED, SSCI, A&HCI, CPCI-S, CPCI-SSH, BKCI-S, BKCI-SSH, ESCI, CCR-EXPANDED, IC Timespan=All years* | [Edit](http://apps.webofknowledge.com/WOS_AdvancedSearch_input.do?product=WOS&SID=C56HEtGVniJkJ8gFEJW&search_mode=AdvancedSearch&replaceSetId=16&editState=init) |  |  |
|  | | | | | |
| # 15 | [**16,991**](http://apps.webofknowledge.com/summary.do?product=WOS&doc=1&qid=15&SID=C56HEtGVniJkJ8gFEJW&search_mode=GeneralSearch&update_back2search_link_param=yes) | **TOPIC:** (childbirth)  *Indexes=SCI-EXPANDED, SSCI, A&HCI, CPCI-S, CPCI-SSH, BKCI-S, BKCI-SSH, ESCI, CCR-EXPANDED, IC Timespan=All years* | [Edit](http://apps.webofknowledge.com/WOS_AdvancedSearch_input.do?product=WOS&SID=C56HEtGVniJkJ8gFEJW&search_mode=AdvancedSearch&replaceSetId=15&editState=init) |  |  |
|  | | | | | |
| # 14 | [**67,032**](http://apps.webofknowledge.com/summary.do?product=WOS&doc=1&qid=14&SID=C56HEtGVniJkJ8gFEJW&search_mode=GeneralSearch&update_back2search_link_param=yes) | **TOPIC:** (perinatal)  *Indexes=SCI-EXPANDED, SSCI, A&HCI, CPCI-S, CPCI-SSH, BKCI-S, BKCI-SSH, ESCI, CCR-EXPANDED, IC Timespan=All years* | [Edit](http://apps.webofknowledge.com/WOS_AdvancedSearch_input.do?product=WOS&SID=C56HEtGVniJkJ8gFEJW&search_mode=AdvancedSearch&replaceSetId=14&editState=init) |  |  |
|  | | | | | |
| # 13 | [**4,249**](http://apps.webofknowledge.com/summary.do?product=WOS&doc=1&qid=13&SID=C56HEtGVniJkJ8gFEJW&search_mode=GeneralSearch&update_back2search_link_param=yes) | **TOPIC:** (puerperal)  *Indexes=SCI-EXPANDED, SSCI, A&HCI, CPCI-S, CPCI-SSH, BKCI-S, BKCI-SSH, ESCI, CCR-EXPANDED, IC Timespan=All years* | [Edit](http://apps.webofknowledge.com/WOS_AdvancedSearch_input.do?product=WOS&SID=C56HEtGVniJkJ8gFEJW&search_mode=AdvancedSearch&replaceSetId=13&editState=init) |  |  |
|  | | | | | |
| # 12 | [**53,321**](http://apps.webofknowledge.com/summary.do?product=WOS&doc=1&qid=12&SID=C56HEtGVniJkJ8gFEJW&search_mode=GeneralSearch&update_back2search_link_param=yes) | **TOPIC:** (postpartum)  *Indexes=SCI-EXPANDED, SSCI, A&HCI, CPCI-S, CPCI-SSH, BKCI-S, BKCI-SSH, ESCI, CCR-EXPANDED, IC Timespan=All years* | [Edit](http://apps.webofknowledge.com/WOS_AdvancedSearch_input.do?product=WOS&SID=C56HEtGVniJkJ8gFEJW&search_mode=AdvancedSearch&replaceSetId=12&editState=init) |  |  |
|  | | | | | |
| # 11 | [**104,158**](http://apps.webofknowledge.com/summary.do?product=WOS&doc=1&qid=11&SID=C56HEtGVniJkJ8gFEJW&search_mode=GeneralSearch&update_back2search_link_param=yes) | **TOPIC:** (postnatal)  *Indexes=SCI-EXPANDED, SSCI, A&HCI, CPCI-S, CPCI-SSH, BKCI-S, BKCI-SSH, ESCI, CCR-EXPANDED, IC Timespan=All years* | [Edit](http://apps.webofknowledge.com/WOS_AdvancedSearch_input.do?product=WOS&SID=C56HEtGVniJkJ8gFEJW&search_mode=AdvancedSearch&replaceSetId=11&editState=init) |  |  |
|  | | | | | |
| # 10 | [**2,380,152**](http://apps.webofknowledge.com/summary.do?product=WOS&doc=1&qid=10&SID=C56HEtGVniJkJ8gFEJW&search_mode=CombineSearches&update_back2search_link_param=yes) | #9 OR #8 OR #7 OR #6 OR #5 OR #4 OR #3 OR #2 OR #1  *Indexes=SCI-EXPANDED, SSCI, A&HCI, CPCI-S, CPCI-SSH, BKCI-S, BKCI-SSH, ESCI, CCR-EXPANDED, IC Timespan=All years* | [Edit](http://apps.webofknowledge.com/WOS_AdvancedSearch_input.do?product=WOS&SID=C56HEtGVniJkJ8gFEJW&search_mode=AdvancedSearch&replaceSetId=10&editState=init) |  |  |
|  | | | | | |
| # 9 | [**19,228**](http://apps.webofknowledge.com/summary.do?product=WOS&doc=1&qid=9&SID=C56HEtGVniJkJ8gFEJW&search_mode=GeneralSearch&update_back2search_link_param=yes) | **TOPIC:** (new mothers)  *Indexes=SCI-EXPANDED, SSCI, A&HCI, CPCI-S, CPCI-SSH, BKCI-S, BKCI-SSH, ESCI, CCR-EXPANDED, IC Timespan=All years* | [Edit](http://apps.webofknowledge.com/WOS_AdvancedSearch_input.do?product=WOS&SID=C56HEtGVniJkJ8gFEJW&search_mode=AdvancedSearch&replaceSetId=9&editState=init) |  |  |
|  | | | | | |
| # 8 | [**1,308,738**](http://apps.webofknowledge.com/summary.do?product=WOS&doc=1&qid=8&SID=C56HEtGVniJkJ8gFEJW&search_mode=GeneralSearch&update_back2search_link_param=yes) | **TOPIC:** (woman)  *Indexes=SCI-EXPANDED, SSCI, A&HCI, CPCI-S, CPCI-SSH, BKCI-S, BKCI-SSH, ESCI, CCR-EXPANDED, IC Timespan=All years* | [Edit](http://apps.webofknowledge.com/WOS_AdvancedSearch_input.do?product=WOS&SID=C56HEtGVniJkJ8gFEJW&search_mode=AdvancedSearch&replaceSetId=8&editState=init) |  |  |
|  | | | | | |
| # 7 | [**1,308,738**](http://apps.webofknowledge.com/summary.do?product=WOS&doc=1&qid=7&SID=C56HEtGVniJkJ8gFEJW&search_mode=GeneralSearch&update_back2search_link_param=yes) | **TOPIC:** (women)  *Indexes=SCI-EXPANDED, SSCI, A&HCI, CPCI-S, CPCI-SSH, BKCI-S, BKCI-SSH, ESCI, CCR-EXPANDED, IC Timespan=All years* | [Edit](http://apps.webofknowledge.com/WOS_AdvancedSearch_input.do?product=WOS&SID=C56HEtGVniJkJ8gFEJW&search_mode=AdvancedSearch&replaceSetId=7&editState=init) |  |  |
|  | | | | | |
| # 6 | [**233,194**](http://apps.webofknowledge.com/summary.do?product=WOS&doc=1&qid=6&SID=C56HEtGVniJkJ8gFEJW&search_mode=GeneralSearch&update_back2search_link_param=yes) | **TOPIC:** (mothers)  *Indexes=SCI-EXPANDED, SSCI, A&HCI, CPCI-S, CPCI-SSH, BKCI-S, BKCI-SSH, ESCI, CCR-EXPANDED, IC Timespan=All years* | [Edit](http://apps.webofknowledge.com/WOS_AdvancedSearch_input.do?product=WOS&SID=C56HEtGVniJkJ8gFEJW&search_mode=AdvancedSearch&replaceSetId=6&editState=init) |  |  |
|  | | | | | |
| # 5 | [**233,194**](http://apps.webofknowledge.com/summary.do?product=WOS&doc=1&qid=5&SID=C56HEtGVniJkJ8gFEJW&search_mode=GeneralSearch&update_back2search_link_param=yes) | **TOPIC:** (mother)  *Indexes=SCI-EXPANDED, SSCI, A&HCI, CPCI-S, CPCI-SSH, BKCI-S, BKCI-SSH, ESCI, CCR-EXPANDED, IC Timespan=All years* | [Edit](http://apps.webofknowledge.com/WOS_AdvancedSearch_input.do?product=WOS&SID=C56HEtGVniJkJ8gFEJW&search_mode=AdvancedSearch&replaceSetId=5&editState=init) |  |  |
|  | | | | | |
| # 4 | [**127,531**](http://apps.webofknowledge.com/summary.do?product=WOS&doc=1&qid=4&SID=C56HEtGVniJkJ8gFEJW&search_mode=GeneralSearch&update_back2search_link_param=yes) | **TOPIC:** (mums)  *Indexes=SCI-EXPANDED, SSCI, A&HCI, CPCI-S, CPCI-SSH, BKCI-S, BKCI-SSH, ESCI, CCR-EXPANDED, IC Timespan=All years* | [Edit](http://apps.webofknowledge.com/WOS_AdvancedSearch_input.do?product=WOS&SID=C56HEtGVniJkJ8gFEJW&search_mode=AdvancedSearch&replaceSetId=4&editState=init) |  |  |
|  | | | | | |
| # 3 | [**127,531**](http://apps.webofknowledge.com/summary.do?product=WOS&doc=1&qid=3&SID=C56HEtGVniJkJ8gFEJW&search_mode=GeneralSearch&update_back2search_link_param=yes) | **TOPIC:** (mum)  *Indexes=SCI-EXPANDED, SSCI, A&HCI, CPCI-S, CPCI-SSH, BKCI-S, BKCI-SSH, ESCI, CCR-EXPANDED, IC Timespan=All years* | [Edit](http://apps.webofknowledge.com/WOS_AdvancedSearch_input.do?product=WOS&SID=C56HEtGVniJkJ8gFEJW&search_mode=AdvancedSearch&replaceSetId=3&editState=init) |  |  |
|  | | | | | |
| # 2 | [**942,614**](http://apps.webofknowledge.com/summary.do?product=WOS&doc=1&qid=2&SID=C56HEtGVniJkJ8gFEJW&search_mode=GeneralSearch&update_back2search_link_param=yes) | **TOPIC:** (females)  *Indexes=SCI-EXPANDED, SSCI, A&HCI, CPCI-S, CPCI-SSH, BKCI-S, BKCI-SSH, ESCI, CCR-EXPANDED, IC Timespan=All years* | [Edit](http://apps.webofknowledge.com/WOS_AdvancedSearch_input.do?product=WOS&SID=C56HEtGVniJkJ8gFEJW&search_mode=AdvancedSearch&replaceSetId=2&editState=init) |  |  |
|  | | | | | |
| # 1 | [**942,614**](http://apps.webofknowledge.com/summary.do?product=WOS&doc=1&qid=1&SID=C56HEtGVniJkJ8gFEJW&search_mode=GeneralSearch&update_back2search_link_param=yes) | **TOPIC:** (female)  *Indexes=SCI-EXPANDED, SSCI, A&HCI, CPCI-S, CPCI-SSH, BKCI-S, BKCI-SSH, ESCI, CCR-EXPANDED, IC Timespan=All years* | [Edit](http://apps.webofknowledge.com/WOS_AdvancedSearch_input.do?product=WOS&SID=C56HEtGVniJkJ8gFEJW&search_mode=AdvancedSearch&replaceSetId=1&editState=init) |  |  |
|  | | | | | |
|  |  |  |  | AND   OR  **Combine** | **Select All  Delete** |

Bottom of Form
